# Exploring story grammar structure in the book reading interactions of African American mothers and their preschool children: a pilot investigation

**DOI:** 10.3389/fpsyg.2014.00545

**Published:** 2014-06-04

**Authors:** Yvette R. Harris, Susan E. Rothstein

**Affiliations:** ^1^Department of Psychology, Miami UniversityOxford, OH, USA; ^2^Psychology, Miami UniversityOxford, OH, USA

**Keywords:** African American mothers and children, book reading interactions, home reading environment, book reading styles, story grammar use and African American mothers

## Abstract

The aim of this investigation was to identify the book reading behaviors and book reading styles of middle class African American mothers engaged in a shared book reading activity with their preschool children. To this end, the mothers and their children were videotaped reading one of three books, Julius, Grandfather and I, or Somewhere in Africa. Both maternal and child behaviors were coded for the frequency of occurrence of story grammar elements contained in their stories and maternal behaviors were also coded for their use of narrative eliciting strategies. In addition, mothers were queried about the quality and quantity of book reading/story telling interactions in the home environment. The results suggest that there is a great deal of individual variation in how mothers use the story grammar elements and narrative eliciting strategies to engage their children in a shared book reading activity. Findings are discussed in terms of suggestions for additional research and practical applications are offered on ways to optimally engage African American preschool children and African American families from diverse socioeconomic backgrounds in shared book reading interactions.

## Introduction

For the past several decades, literacy researchers have turned their attention to exploring the ways in which mothers' structure and guide story telling interactions and shared book reading interactions with their children. As a consequence of this interest, a rich body of research has emerged identifying the different types of maternal conversational styles, book reading styles or story telling styles, and at some level correlating those styles with their children's immediate and long-term cognitive and literacy performances. This research has differed with respect to operationalization of maternal conversational styles and storytelling styles, and definition of narrative context. For example, Fivush and Fromhoff (1988) explored the conversational styles that mothers used to engage their children in a narrative context which involved conversations about the past. They observed that mothers employed either an elaborative conversational style or a repetitive conversational style. In the former style, mothers asked questions, re-casted statements, and provided fill in the blank details to their children's responses. In the latter style mothers mainly asked questions to elicit recall from their children and rarely varied their use of strategies. Children of mothers who employed an elaborate conversational style recalled more information about the past event in comparison to children whose mothers employed a repetitive conversational style.

Continuing along this line of research, and using a similar narrative context, Haden et al. (1997) found that maternal conversational styles fell into three distinct categories. The first category consisted of mothers classified as describers and these mothers used strategies that defined the past event, and strategies that elaborated on the past event. The second category was comprised of mothers who were identified as collaborators. They used strategies which encouraged their children to co-tell the event with them. Lastly, there were mothers who were classified as comprehenders. These mothers employed strategies that prompted their children to make connections with present experiences and past experiences. Haden et al. concluded that children of mothers who employed the latter two styles were more accurate in their recall of the past event in comparison to children whose mothers were classified as describers.

Other researchers have investigated maternal narrative styles or conversational styles as they emerged in the context of shared book reading activities with their children. Wellborn et al. (1999) identified two different maternal narrative styles in their study; story collaborators and story tellers. The story collaborators used strategies such as questions and prompt to engage their children in the book reading activity; whereas as the story tellers primarily used statements to engage their children in the book reading activity. According to Wellborn et al. (1999) children whose mothers used a story collaborative style were more involved in the interaction than were children whose mothers employed a story teller style. In a slightly similar study, Hammer et al. (2005) observed four distinct maternal narrative styles. There were mothers who were classified as text readers. Text readers read verbatim from the book without making requests to include their children in the reading activity. By contrast, some mothers were identified as labelers and these mothers read each word, inserting pauses between each word, and similar to text readers made little effort to encourage their children to participate in the book reading activity. A few mothers were classified as child centered and these mothers allowed their children to assume control in the book reading interaction. Lastly Hammer et al. (2005) observed that some mothers used a combination style. In this case, they incorporated strategies reflective of the other three styles. Hammer et al. noted that children whose mothers used a child centered or combination narrative style were more involved in the book reading activity.

Using a fairly different method, Harris and Schroeder (2011) investigated maternal story telling styles as they occurred in the context of a constructive play activity. In this particular study, mothers were asked to tell their children a story about the objects/characters involved in the play activity and have their children re-tell the story to them. The researchers employing a story grammar schema to evaluate mothers' statements and strategies found that that their story telling styles and narrative eliciting styles cohered into two distinct categories. Thirty eight percent of the mothers used an inclusive story grammar style, and 62 percent used a restrictive story grammar style. Mothers using an inclusive story grammar style spent most of their time providing detailed instructions on the nature of the activity, providing detailed descriptions of the actions of the characters, and conversations between the characters; whereas mothers employing a restrictive story grammar style gave a brief overview of the goals of the activity, and spent most of their time discussing the location of the characters. Children of mothers, who employed the former narrative style were more engaged in the play activity as evidenced by their questions and comments during the interaction. With reference to narrative eliciting strategies, 16 percent of the mothers were identified as narrative scaffolders and 84 percent were identified as narrative solicitors. Narrative scaffolders elicited recall from their children by asking questions, using prompting statements, and providing corrective feedback to their children. In contrast, narrative solicitors spent most of their time eliciting recall about the story from their children through a series of prompting questions and provided little feedback to their children about their performance. Children of mothers identified as narrative scaffolders were able to retell more of the story about the play activity in comparison to children of mothers who were classified as narrative solicitors.

Collectively this corpus of research demonstrates that there is a great deal of variation in maternal narrative styles, and these styles differ to some extent depending on narrative context. Furthermore there is evidence to suggest that styles that invite children in the narrative activity appear to promote their active engagement.

In addition to examining maternal stylistic differences in narrative, play, and book reading activities, literacy researchers have also explored the quality of the book reading and literacy environment of the home. The comprehensive findings from this body of research suggests that literacy artifacts (books, newspapers), functional uses of literacy (reading), parental attitudes about literacy especially reading, occur with varying frequency in the homes of preschool children (Phillips and Lonigan, 2009). That is there are cultural and socioeconomic status differences with respect to the frequency with which parents provide their children with access to literacy artifacts, and engage in literacy related behaviors in the home environment with their children (Leseman and de Jong, 1998; Frijters et al., 2000; Burgess et al., 2002).

Only a few studies to date have looked at the conversational, book reading, or narrative styles of African American mothers engaged in a variety of narrative activities with their children. Those studies which do exist have focused mainly on low income African American mothers and their book reading interactions with their infants and toddlers (Pelligrini et al., 1999; Hammer et al., 2005). While these studies have provided literacy researchers with a wealth of information on the book reading behaviors and book reading styles of low income African American mothers, they yield little information on the book reading behaviors of middle class African American mothers. This is problematic for several reasons. First, the findings from the research on low income African American mothers are frequently generalized to account for the behaviors and practices of African American middle class mothers and as such researchers have an inaccurate and incomplete picture of the literacy practices and behaviors which occur in an economically diverse sample of African American mothers and their children. Second, as Heath (1983) and Tamis-LeMonda et al. (2008), state an area as critical as literacy should include explorations of a diverse array of cultural and economic groups; as culture broadly and narrowly defined plays a pivotal role in children's access to literacy materials, interactions with literacy materials, and subsequently their literacy performance in a formal academic setting. This gap in the literature serves as the major impetus for this present investigation.

This study is designed to address the following research questions.

Our first research question explores the book reading behavior of African American mothers engaged in a shared book reading activity with their children. We operationalize maternal book reading behavior in several ways. First, we examine their use of story grammar elements. Story grammar refers to the internal structure of simple stories or narratives. This internal structure frequently involves a beginning, settings, character descriptions, goals, actions, consequences, dialog, internal responses, and endings (Mandler and Johnson, 1977). Research indicates that stories which cohere to a formula that contains a beginning, middle, and ending are easily understood and recalled better by both adults and children (McCabe and Peterson, 1991). While books are traditionally designed to follow a story grammar structure, we are interested in determining the frequency with which mothers' emphasize those aspects of story grammar structure in their book reading behavior. Second, we examine their use of narrative eliciting strategies as they engage their children in the book reading activity. Narrative eliciting strategies as defined by Harris and Schroeder (2011) are questions, prompts, and statements employed by mothers to maintain and involve their children in the book reading activity.

Our second research question examines how preschool children participate in the book reading activity with their mothers. Our specific interest is to explore the story grammar elements present in the preschoolers' vocalizations about the story. That is, do they use the same story grammar elements that mothers use? Researchers have found a correspondence between the verbalizations that mothers use in their narratives, and the verbalizations that children use as they re-tell stories (Haden et al., 1997).

Our third question investigates the quality of book reading-type activities available to the preschool children in their home environment. Using the Parental Support for Reading Activities in the Home Environment we queried mothers about their children's access to print, reading activities, and reading interactions in the home environment. We are also interested in determining the relationship between maternal book reading style and the quality of their home book reading environment.

In summary, the goal of this pilot project was to examine the book reading interactions of African American mothers and their preschool children. Mothers were observed engaged in a shared book reading activity with their preschool children, and a story grammar schema was used to identify their specific book reading strategies and book reading styles. We were also interested in determining the story grammar elements present in the preschoolers' verbalizations about the stories. In addition we asked mothers to respond to questions about the types of book reading interactions and activities available to their children in the home environment.

## Materials and methods

### Partcipants

Eight African American mothers and their preschool children recruited from childcare centers in a tri-county area participated in the study. Four of the children were males, and four were females. The children ranged in from 36 to 60 months (*M* = 54 month, *SD* = 7.1). The mothers on average were 30 years of age, married, and college educated. They were offered a payment of ten dollars for their participation in the research, and the children were offered a small toy.

### Stimuli

#### Story books

Three story books were used as the stimuli in this present investigation: *Julius* (Johnson, 1993) which is a story about a young pig who lives with Mia and her parents; *Grandfather and I* (Buckely and Ormerod, 1994) is a story which focuses on the relationship between an African American grandfather and his grandson as they go about their daily activities; and *Somewhere in Africa* (Mennen and Daly, 1997) depicts the travels of a young boy as he journeys throughout Africa. The books for the most part are of equal length, contain the same narrative structure; all are illustrated and portray the lives of African or African American children and their families in central plot lines.

*Parental Support for Reading Activities in the Home Environment Questionnaire* is an 11 item questionnaire divided into two sections. The first section entitled, *Parental and Child Reading Interactions* contains questions that ask mothers to provide information on how frequently they engage in book reading activities in the home with their children. Examples of questions include: 1) How frequently do you read to you child? 2) How frequently does your child asked to be read to? 3) How frequently do you discuss reading material with their child? Answers to these questions were scored on a 5 point scale ranging from 5 (daily) to 1 (never). Additional questions asked mothers to provide information indicating whether their children had a regular reading time, at what age did they begin reading to their child, how frequently the child tries to read to them, to identity family members who read to the child, and how frequently is storytelling without a book a regular family activity?

The second section, *Support for Reading Activities in the Home Environment* is comprised of questions which probe mothers about the ways in which they provide support for reading related activities in the home environment. Examples include: (1) Does your child have any magazine or book subscriptions? (2) How many books are in your home? (3) Do you purchase games to help the child learn to read? (4) Does your child have a library card? (5) Does your child check out books from the library? Answers to these questions were coded yes or no.

Mothers were also asked to provide basic demographic information. Copies of the full questionnaire may be obtained from the investigators upon request.

#### Procedure

Upon arrival to the testing site, the mothers first completed the Parental Support for Reading Activities in the Home Environment Questionnaire. After completion of the questionnaire, the mothers were then videotaped reading one of three books to their children. The mothers were instructed by the researcher to read the books to their children in a way that they were comfortable with, there were no time constraints placed on the session and they were free to determine when to terminate the session.

#### Coding system

A modification of the Harris and Schroeder Story Grammar Coding System (2011) was used to code maternal and child story grammar elements and maternal narrative eliciting strategies. The videotapes were first transcribed and the transcripts were then coded by undergraduate psychology research assistants blind to the research questions of the study. The behaviors were coded for their frequency of occurrence.

#### Maternal (M) child (C) story grammar elements

**Table:** 

Strategy	Description
Story beginnings (MSB/CSB)	Verbal indication that the story has begun
Settings (MST/CST)	Verbal references to the location, background, or time frame of the story
Naming/labeling (MNL/CNL)	Simple naming or labeling of the objects, characters in the story
Character descriptions(MCD/CCD)	Verbal references to the names, physical appearances and social roles of the characters in the book
Goals (MG/CG)	Verbal reference to the purpose, plans, intentions, wants, or desires of one or more of the characters in the book
Actions (MA/CA)	Verbal reference regarding a series of actions carried out by one or more of the characters in the book
Actions (MA/CA)	Verbal reference regarding a series of actions carried out by one or more of the characters in the book
Consequences (MC/CC)	Verbal references to direct consequence of an action carried out by one or more of the characters in the book
Consequences (MC/CC)	Verbal references to direct consequence of an action carried out by one or more of the characters in the book
Dialog (MD/CD)	Verbal reference to conversation between characters in the form of quoted speech or implied dialog in the book
Dialog (MD/CD)	Verbal reference to conversation between characters in the form of quoted speech or implied dialog in the book
Internal responses(MIR/CIR)	Verbal reference to the inner thoughts or emotions of one or more of the characters in the book
Internal responses(MIR/CIR)	Verbal reference to the inner thoughts or emotions of one or more of the characters in the book
Endings (ME/CE)	Verbal reference indicating that the story has ended
Endings (ME/CE)	Verbal reference indicating that the story has ended

#### Maternal narrative eliciting strategies

**Table:** 

**Strategy**	**Description**
Sequential questions (SQ)	Questions used to elicit the order of events in the story (e.g., what comes next, did this happen next?)
General question (GQ)	Questions used to elicit information about the names of the characters, character actions, feelings and relationships (e.g., who is this, what did Julius do? Where was Grandfather going? Where does Ashraf live?)
Re-focusing statements (RF)	Statements that refocus the child's attention to the story when the child strays off task (e.g., look at me)
Re-tells the story (RTS)	Statements that retell all or part of the story (e.g., let me tell my story again)
Re-casting (RC)	Statements that re-state the child's story or sentence to make it consistent with her story
Encourage (E)	Good job, you can do it
Fill in the blank statements (FIBS)	Statements that complete the child's story
Prompting statements (PS)	Statements such as did this happen, now can you tell me the story I just told you
Reference to memory (RFM)	Statements referring to the child's memory (e.g., can you remember what I just said? Come on you can remember it?)

### Reliability

Two raters independently coded 20 percent of the transcripts for reliability. Percentage of agreement was used as the reliability estimate and there was 90 percent reliability agreement among the raters for the strategies. Any disagreements were resolved through discussion. The remainder of the transcripts were divided and coded independently by the raters.

## Results

The results section begins with a discussion of the Parental Support for Reading Activities in the Home Environment, continues with a presentation of the descriptive statistics on the story grammar elements and narrative eliciting strategies present in the mothers' book reading and with a discussion of the story grammar elements present in the children's vocalizations about the stories. The section concludes with a discussion of maternal book reading styles.

### Parental support for reading activities in the home environment

Descriptive statistics for the home environment questionnaire are presented in Table 1. As the data in the table indicate, 83% of the mothers report reading to their children daily; and over half of the children frequently solicit requests to be read to from their mothers or from other family members. Fifty percent of the children have a regular reading time, over half of the mothers report they frequently engage in discussing reading materials with their children and the majority of the children have a favorite book. Over half of the children have books and or magazine subscriptions; and sixty-six percent of the mothers purchase specific material to help their children learn to read. The majority of the mothers report that their children have a library card and all report that their children check out books from the library.

**Table 1:** **Parental support for reading activities in the home environment**.

**Questions**	**Percent**
**PARENTAL AND CHILD BOOK READING INTERACTIONS**	
How frequently do you read to your child?	83% Daily
How frequently does your child asked to be read to?	83% Daily
How frequently do you engage in discussing reading material with your child?	83% Daily
Does your child have a favorite book?	83% Yes
Is there a regular reading time?	50% Yes
	50% No
Does any other family member read to the child?	100% Yes
**SUPPORT FOR BOOK READING ACTIVITIES IN THE HOME**	
Does your child have a magazine/book subscription?	50% Yes
Do your purchase reading material?	66% Yes
Do you purchase reading games?	83% Yes
Does your child have a library card?	83% Yes
Does your child check out books from the library?	100% Yes

### Story grammar elements present in mothers' book reading

**Figure 1:**
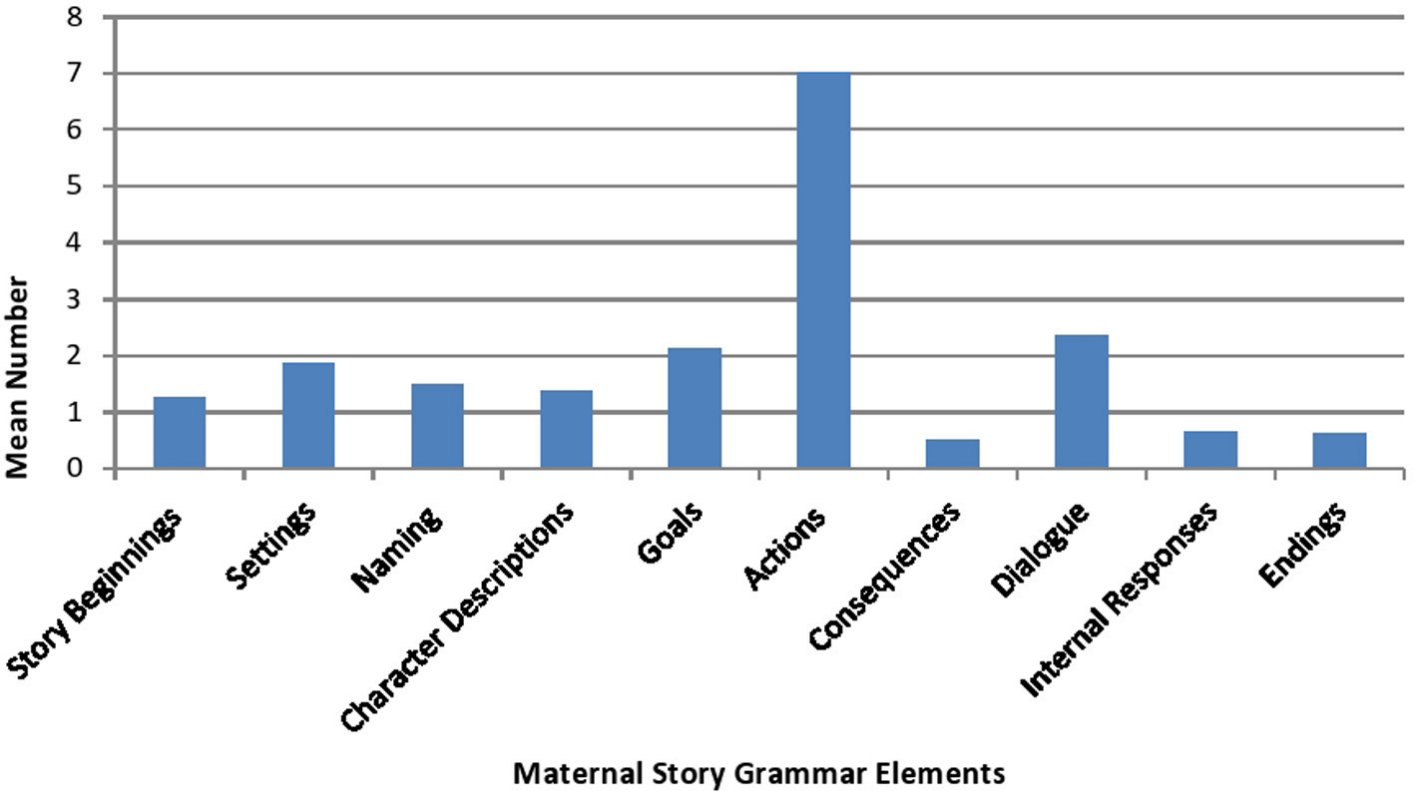
**Mean number of maternal story grammar elements**.

Figure 1 provides information on the mean number of story grammar elements present in the mothers' book reading. As the figure shows, mothers used a variety of story grammar elements while reading the books to their children, however they placed special attention on discussing the actions of the characters (*M* = 7, *SD* = 4.5) and the dialog between characters, (*M* = 2.3, *SD* = 1.9), they mentioned the wants and desires of the characters in the story (*M* = 2.1, *SD* = 3.6), highlighted the settings of the story (*M* = 1.87, *SD* = 1.64), and stressed the social roles and the physical appearances of the characters in the book (*M* = 1.37, *SD* = 0.74).

### Narrative eliciting strategies present in mothers' book reading

**Figure 2:**
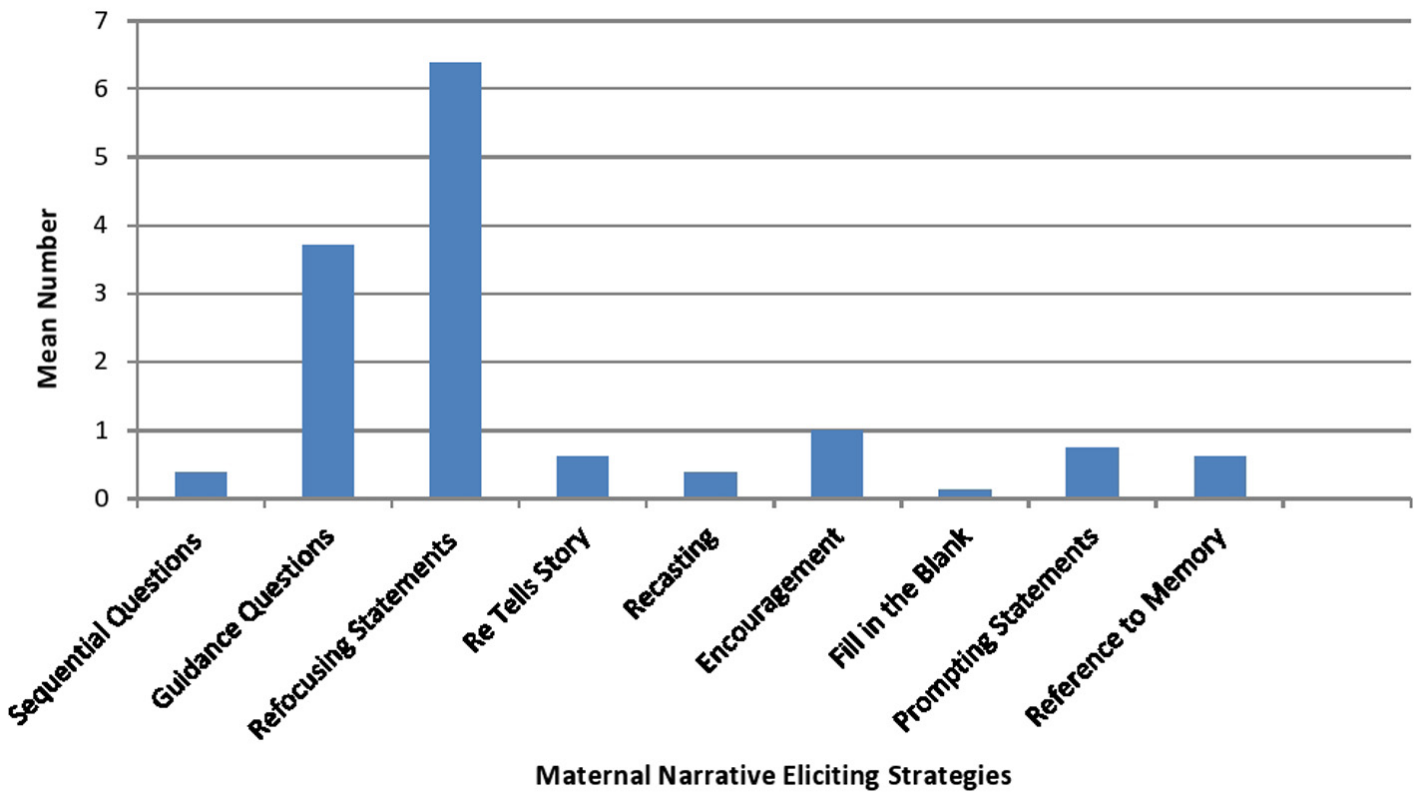
**Mean number of maternal narrative eliciting strategies**.

Figure 2 presents the means on the narrative eliciting strategies used by mothers to engage their children in the book reading interaction. As the figure illustrates, while mothers used an assortment of narrative eliciting strategies, they most often employed refocusing statements to elicit engagement in the interaction from their children (*M* = 6.3, *SD* = 7.65), asked general questions about the characters in the story book (*M* = 3.7; *SD* = 2.25), and frequently encouraged their children's vocalizations during the book reading activity (*M* = 1, *SD* = 1.19).

### Preschooler story grammar use during the book reading activity

**Figure 3:**
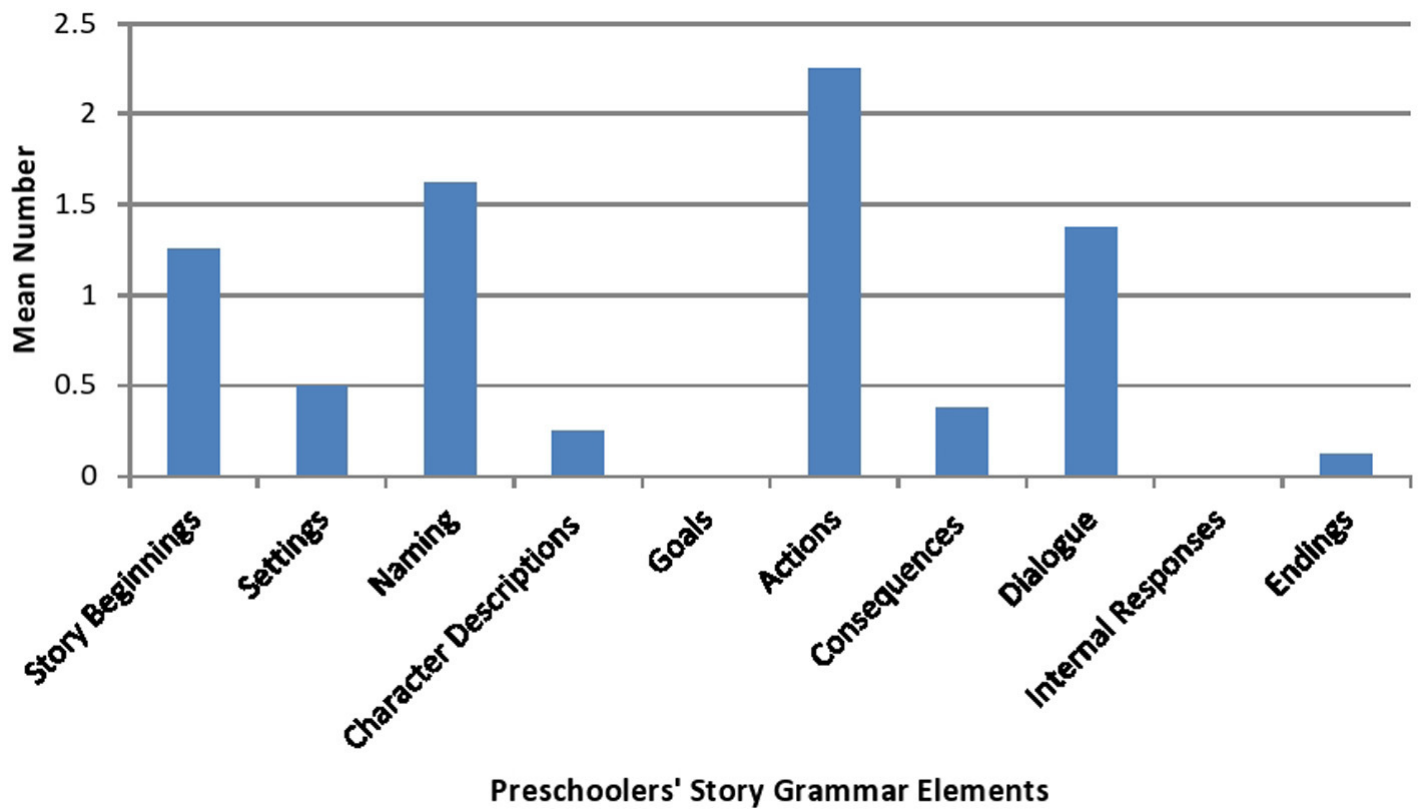
**Mean number of preschoolers' story grammar elements**.

Figure 3 depicts the means for the preschoolers' story grammar use during the book reading interaction. While they used considerably fewer story grammar elements than their mothers, they engaged in the book reading interaction by commenting on the actions carried out by the characters (*M* = 2.2, *SD* = 2.18), generating the names of the characters of the book (*M* = 1.6, *SD* = 1.99), and remarking on the dialog or conversations occurring between the characters (*M* = 1.3, *SD* = 1.9).

### Maternal book reading styles

To determine maternal book reading styles a k-means cluster analysis was computed on the maternal story grammar frequencies and the maternal narrative eliciting strategies frequencies. Based on the findings from our previous research, we pre specified two clusters (Harris and Schroeder, 2011). The results of the k-mean cluster analysis for this study revealed that thirty eight percent of the mothers were in cluster one, and identified as employing a *modified text focused book* reading style and this style was characterized by an overall greater use of story grammar elements and narrative eliciting strategies. Sixty two percent of the mothers were in cluster two, and classified as using a *text engager* book reading style and characterized by using fewer story grammar elements and narrative eliciting strategies while reading the books to their children.

### Modified text focused book reading style

We classified these mothers as employing a modified text focused book reading style for the following reasons. First, they adhered to a story grammar structure while reading. Second, they modified that structure to include narrative eliciting strategies. The following excerpt is an example of the pattern of interaction.

#### Story beginnings

Mother: This is called Grandfather and I. Sound interesting. Hmm. Grandfather and I are going for a walk. It will be a slow walk because Grandfather and I never hurry. We walk along and stop and look just as long as we like.

Child: Hurry, Hurry

#### Story settings

Mother: Ashraf lives in Africa, not Africa where lions laze in golden grass, not Africa where crocodiles glide through muddy rivers silent a hungry not Africa where Zebra's gallop over Great Plains, Asraf lives in a city at the very tip of the great African content.

Mother: Continues reading. In summer, the city lies soaked in African sun, dry under endless blue sky. Ashraf lives in the city. Where does Ashraf live?

Child: Points to page

#### Character actions

Mother: Turns the page umhmm, Hurry. But grandfather and I never hurry. Look (points at the page). We walk along and walk along. That could be a little Christmas tree. Father hurries off to work and hurry home again. Does your father hurry?

Child: Nods and says sometimes he does?

#### Refocusing statements

Mother: Look J look at the book. Julius made too much noise. Come on Sweetie.

Child: (Stops his attempts to get off the chair).

Mother: Look at the hats. Which one is red?

Child: (points to the hat and smiles). That one.

### Text engager book reading style

We labeled these mothers as using a text engager book reading style for the following reasons. One, they used fewer story grammar elements in reading the book to their children and two, they began their reading with narrative eliciting strategies and continued to use these strategies throughout their reading. The example below illustrates the pattern of interaction between these mothers and their children.

#### They began their stories by naming the characters and continued the book reading activity by asking questions

Mother: It says somewhere in Africa. You see that? See the picture? He's got a book in his hand.

Child: Like us?

Mother: Mm Hm Begins to read the book.

Mother: (Stops, and asks) what's that right here?

Child: Airplane.

Mother: Good…. Continues reading. Not Africa where crocodiles glide through the muddy rivers. See them? The crocodiles?

Child: They came to eat the boy?

Mother: They are not going to eat the boy.

Child: Uh Huh!!

Mother: Continues Reading. Look at the Zebra's

Child: They run from somebody?

Mother: No.

#### Used fill in the blank statements to complete the child's story

Mother: Who is this? What is her name?

Child: I don't know… …

Mother: Mia and who might these people be?

Child: Father

Mother: Father and

Child: Mommy

#### Recasts the child's statements to be consistent with hers

Mother: Mia's parents did think they would like Julius. He showed them no fun and no sharing. Mia loved Julius. Why didn't Mia's parents like Julius?

Child: He's no fun?

Mother: He showed them no fun and no sharing.

We evaluated the Parental Support for Reading Activities in the Home Environment to determine if there might be differences in the home environments of modified text focused mothers and the text engager mothers. No significant differences were observed.

## Discussion

The general goal of this research was to explore the ways in which middle class African American mothers' structure and guide a book reading interaction for their preschool children. To accomplish this, we examined the presence of story grammar elements in their book reading, the types of narrative eliciting strategies they employed to maintain their children's attention during the book reading activity, and queried them about the quantity and quality of book reading activities and interactions available to their children in their home environment. In addition, we identified the types of story grammar elements present in the preschoolers vocalizations about the stories.

Our first finding suggests that middle class African American mothers use a variety of story grammar elements while reading books to their children. That is, they emphasize the actions of the characters, they engage in some form of character dialog, and they reference the goals of the story. To elicit contributions to the book reading interaction from their children, they employ refocusing statements, ask general questions, and encourage the children's participation. The preschoolers, while they produce fewer vocalizations about the books than their mothers, similar to their mothers, commented on the actions of the characters and remarked on the dialog taking place between the characters.

Our second finding reveals that there are book reading styles that distinguish these mothers. A small percentage of the mothers in our study employed a modified text focused book reading style. In this instance, they began the story with an orientation indicating that the story had begun, and continued to focus on such salient aspects of the story such as setting, goals and descriptions of the actions of the characters in the story. While it is difficult to directly compare these styles to the styles other literacy researchers have observed; and that was not the intent of our research, we speculate that this style may be similar to the text reader style identified by Hammer et al. (2005). However, our mothers differ in that they rarely read directly from the book and they modified their reading to include strategies to re-engage their children in the reading activity. The majority of the mothers in our study used a text engager book reading style. They began their reading with questions to immediately capture their children's attention in the book reading activity. Their style may be reflective of what Heath (1983) refers to “using the book as a prop style.” Books serve as a prop to facilitate interaction and perhaps an opportunity for the children to gain rudimentary knowledge of the mechanics involved in reading books. Their style also slightly resembles the child centered book reading style identified in the Hammer et al. (2005) research, but is somewhat different in that they did not surrender control of the book reading interaction to their children. They quickly launched into the activity, and employed a series of questions to capture and sustain the children's attention.

Our third finding suggests that African American children reared in middle class homes have print rich experiences and frequently engage in reading interactions and reading activities with their mothers and other family members. There were no differences in the home reading environments of children whose mothers employed the diverse book reading styles. It may be the case that home book reading environments are created to sustain and support the book literacy of all members of the families, as opposed to individual children. Furthermore it might be that differences in the home literacy environment emerge when children enter a formal academic setting.

Clearly there are limitations with our research. The small sample size limits the generalizability of our findings and as such the findings must be interpreted with some degree of caution. Thus any future research must consider increasing the sample size when addressing the following next steps.

There are several important next steps in this line of research. First, future research in this area must take into account the nature of the book reading materials. In contrast to the previous research on shared book reading and African American mothers and their children, we observed mothers reading books to their children consisting of African American characters and storylines. No studies to date have employed such a methodology. However, there is a current focus in U. S. preschool classrooms to introduce African American families to books that depict the lives of African American families and children. One notably example of this effort comes from the recent work of McNair (2014) who developed a family literacy program for an economically diverse group of African American parents and their children. The goals of the program were to introduce these families to books written about African American children, and to provide them with suggestions on ways to create a stimulating home reading environment. According to McNair, the follow up interviews with the parents revealed that many of them reported being unaware of books written with African American children and families as central characters; some stated that as a result of the program they increased their reading time with their children; several indicated that they were sharing their knowledge of African American children's books with other parents and a few reported purchasing additional books with African American children and families as central characters. McNair concluded that in order for African American children to develop as successful readers it is important that African American children “see images” of themselves reflected in literature. Nyhout and O'Neil (2013) underscore this point and state that studies investigating shared book reading interactions “overlook the important role the book itself may play in the interaction and the role the book may play in the preschoolers' engagement in a shared book reading activity” (p. 2).

Subsequent research continuing along this line of study, could explore the influence of text content (i.e., books containing African American children and families as central characters vs. African American children and families absent from text) on maternal book reading strategies and book reading styles and the preschoolers' engagement in the book reading activity.

We also recommend that future investigations employ a microgenetic design (Siegler and Crowley, 1991). It may be the case that maternal book reading styles and strategies change along with book familiarity. In addition, there may be parallel changes which occur in the home book reading environment, that are best captured by a microgenetic design. Lastly, it is important to assess the children's contribution to the book reading interaction as their contribution and participation may change in tandem to maternal behavior.

In conclusion, the findings from this pilot study point toward the need for continued investigation into the book reading interactions of African American mothers and their children from diverse educational and income backgrounds. Based on this work, we have identified stylistic differences in their book reading behavior and captured at some level their children's engagement the book reading activity.

### Conflict of interest statement

The authors declare that the research was conducted in the absence of any commercial or financial relationships that could be construed as a potential conflict of interest.

## References

[B2] Buckely H. Ormerod J. (1994). Grandfather and I. New York, NY: Lothrop, Lee and Shepard Books

[B3] Burgess S. Hecht S. A. Lonigan C. J. (2002). Relations of home literacy environment to the development of reading related abilities: a one year longitudinal study. Read. Res. Q. 40, 408–426 10.1598/RRQ.37.4.4

[B4] Fivush R. Fromhoff F. (1988). Style and Structure in mother child conversation about the past. Dis. Process. 11, 337–355 10.1080/01638538809544707

[B5] Frijters J. C. Barron R. W. Brunello M. (2000). Direct and mediated influences of home literacy and literacy interests on prereaders' oral vocabulary and early written language skill. J. Educ. Psychol. 92, 466–477 10.1037/0022-0663.92.3.466

[B6] Haden C. A. Reese E. Fivush R. (1997). Mothers' extra textual comments during storybook reading. Stylistic changes overtime and across context. Dis. Process. 21, 135–169 10.1080/01638539609544953

[B7a] Hammer C. S. Nimmo D. Cohen R. Draheim H. Johnson A. (2005). Book reading Interactions between African American and Puerto Rican Head Start children and their mothers. J. Early Child. Lit. 5, 195–227 10.1177/1468798405058683

[B8] Harris Y. R. Schroeder V. M. (2011). What the bernstein bears can tell us about school readiness: maternal story grammar and preschool recall. J. Early Childhood Res. 10, 176–195 10.1177/1476718X11430072

[B9] Heath S. (1983). Ways With Words: Language, Life and Work in Communities and Classrooms. New York, NY: Cambridge University Press

[B10] Johnson A. (1993). Julius. New York, NY: Orchard Books

[B11] Leseman P. M. de Jong P. F. (1998). Home literacy: opportunity, instruction cooperation, and social-emotional quality predicting early reading achievement. Read. Res. Q. 33, 294–318 10.1598/RRQ.33.3.3

[B12] Mandler J. Johnson N. (1977). Remembrance of the parsed story structure and recall. Cogn. Psychol. 9, 119–151 10.1016/0010-0285(77)90006-8

[B13] McCabe A. Peterson C. (1991). “Getting the story: a longitudinal study of parental styles in eliciting narratives and developing narrative skill,” in Developing Narrative Structure, eds McCabe A. Peterson C. (Hillsdale, NJ: Erlbaum), 217–253

[B14] McNair J. (2014). “I didn't know there were black cowboys” introducing African American families to African American children's literature. Young Child. 69, 64–69

[B15] Mennen I. Daly N. (1997). Somewhere in Africa. London: Puffin

[B16] Nyhout A. O'Neil D. (2013). Mothers' complex talk when sharing books with their toddlers: book genre matters. First Lang. 33, 115–131 10.1177/0142723713479438

[B17] Pelligrini A. Perlmutter J. Galda L. Brody G. (1999). Joint reading between black head start children and their mothers. Child Dev. 61, 443–453 10.2307/1131106 2344781

[B18] Phillips B. M. Lonigan C. J. (2009). “Social correlates of emergent literacy,” in The Science of Reading: A Handbook, eds Hulme C. Snowling M. (Malden, MA: Blackwell), 173–187

[B21] Siegler R. Crowley P. (1991). The microgenetic method: a direct means for studying cognitive development. Am. Psychol. 46, 606–620 10.1037/0003-066X.46.6.606 1952421

[B22] Tamis-LeMonda C. Briggs R. McClowery S. Snow D. (2008). Challenges to the study of African American parenting: conceptualization, sampling, research approaches, measurement and design. Parent. Sci. Pract. 8, 391–358 10.1080/15295190802612599

[B23] Wellborn A. Thill A. Haden C. (1999). “Mother-child styles of picture book reading,” in *Paper Presented at the Annual Meeting of the Midwestern Psychological Association April* (Chicago, IL).

